# The regulation of host defences to infection by the microbiota

**DOI:** 10.1111/imm.12634

**Published:** 2016-08-09

**Authors:** Rebecca L. Brown, Thomas B. Clarke

**Affiliations:** ^1^ MRC Centre for Molecular Bacteriology and InfectionDepartment of MedicineImperial College LondonLondonUK

**Keywords:** bacterial, inflammation, lung, mucosa, Toll‐like receptors

## Abstract

The skin and mucosal epithelia of humans and other mammals are permanently colonized by large microbial communities (the microbiota). Due to this life‐long association with the microbiota, these microbes have an extensive influence over the physiology of their host organism. It is now becoming apparent that nearly all tissues and organ systems, whether in direct contact with the microbiota or in deeper host sites, are under microbial influence. The immune system is perhaps the most profoundly affected, with the microbiota programming both its innate and adaptive arms. The regulation of immunity by the microbiota helps to protect the host against intestinal and extra‐intestinal infection by many classes of pathogen. In this review, we will discuss the experimental evidence supporting a role for the microbiota in regulating host defences to extra‐intestinal infection, draw together common mechanistic themes, including the central role of pattern recognition receptors, and outline outstanding questions that need to be answered.

## Introduction

The human microbiota is composed of myriad archaea, bacteria, eukaryotes and viruses.^1^, ^2^ It contains approximately 10^13^–10^14^ bacteria that colonize environmentally exposed surfaces.^3^, ^4^ Colonization begins in earnest after birth, with the mother normally providing the bacterial inoculum that seeds the microbiota of her offspring.^4^, ^5^ Initially, the bacterial communities of each surface have a similar taxonomic composition, but through the course of infancy each surface develops a microbiota with a unique composition that stabilizes during adulthood.^2^, ^6^, ^7^ In the adult gastrointestinal tract, the microbiota is dominated by bacteria from two phyla: the Bacteroidetes and Firmicutes.^3^ There are representatives from other bacterial phyla, including Proteobacteria and Acintobacteria, but they form only a minor fraction of the bacterial taxa present.^1^, ^3^ It is thought that many of the members of the Bacteroidetes and Firmicutes are only found within the mammalian gastrointestinal tract, suggesting that these organisms have become specialized to live within this niche and have evolved to form a stable, long‐term interaction with their host.^1^ Other host surfaces colonized by large bacterial communities include the airway and skin.^2^, ^6^ The upper airway has a rich microbial community dominated by bacteria from the Firmicute, Actinobacteria and Proteobacteria phyla,^8^ with the skin being home to mainly Actinobacteria, Proteobacteria and Firmicutes.^2^ Throughout life, diet^9^, ^10^ and host genetics^11^, ^12^ are thought to play a role in shaping microbiota composition, with infection^13^, ^14^, ^15^ and antibiotic treatment^16^, ^17^, ^18^, ^19^ being examples of major causes of microbiota disruption (dysbiosis).

The main host system for interacting with the microbiota is the immune system. At the mucosa the microbiota is in constant, close, contact with both innate and adaptive immune cells.^20^, ^21^, ^22^ This promotes the development and maturation of the mucosal immune system.^20^, ^22^ The resulting agglomeration of immune cells and structures helps to manage and contain the microbiota at the mucosa.^23^, ^24^ The microbiota also drives the maturation of systemic immunity beyond the confines of the mucosa, including in major immune tissues such as the bone marrow and spleen.^25^, ^26^, ^27^, ^28^, ^29^, ^30^ The importance of host–microbiota interactions for our health has been highlighted by clinical studies and work with animal models demonstrating that microbiota dysbiosis is associated with diseases and immune dysfunctions in both intestinal and extra‐intestinal tissues. These include chronic inflammatory conditions in the intestine,^31^, ^32^ autoimmunity^33^, ^34^, ^35^ and increased susceptibility to intestinal and extra‐intestinal infections.^15^, ^30^, ^32^, ^36^, ^37^, ^38^, ^39^, ^40^, ^41^, ^42^, ^43^

The mechanisms by which the microbiota helps to protect against intestinal infection are increasingly well understood. Within the intestine the microbiota stimulates epithelial production of antimicrobial peptides,^44^, ^45^, ^46^ in addition to promoting the antimicrobial activity of immune cells.^47^ These mechanisms of microbiota‐mediated protection against intestinal infection have been extensively reviewed elsewhere^15^, ^38^, ^48^ and will not be discussed in this article. How the microbiota helps to protect against infection at sites outside the intestine (extra‐intestinal), in contrast, is only just becoming clear and is the focus of this review.

## Host resistance to airway infection and the microbiota

Of all extra‐intestinal infections, infection of the respiratory tract is the foremost cause of morbidity and mortality worldwide.^49^ The respiratory tract is a major environmental interface, beginning at the nares of the upper airway extending down to the alveoli in the lower airway (lung). The physiology of the lower airway reflects its role in respiration, with strong pressures to maintain the integrity of the delicate alveoli and to allow efficient gaseous exchange. The lower airway is therefore maintained in a quiescent state to minimize unnecessary inflammation. On occasion, however, microbes that have a commensal lifestyle in the upper airway, including *Streptococcus pneumoniae*,* Staphylococcus aureus* and *Haemophilus influenzae*, do reach the lower airway, often resulting in pneumonia.^8^, ^50^ Lower airway defences must therefore be able to rapidly eliminate these microbes before they cause airway damage and threaten host integrity.

In the lower airway, initial defences are led by the epithelium and alveolar macrophages, the tissue macrophages of the lung.^51^, ^52^, ^53^ Alveolar macrophages perform a variety of functions, including preventing unnecessary inflammation, eliminating pathogens, and restoring lung homeostasis after infection.^54^ As infection proceeds, alveolar macrophages and epithelial defences are bolstered by the recruitment of specialized antimicrobial cells including neutrophils and natural killer cells.^53^ These innate effectors are supplemented by lung dendritic cells that transport antigen to draining lymph nodes for the development of adaptive responses, which are crucial for the clearance of many respiratory pathogens.^53^ Central to the coordination of all these airway defences is initial microbial recognition by pattern recognition receptors (PRRs) leading to the production of a variety of signalling molecules.^52^, ^55^ These include type I interferons during viral infection,^56^ and cytokines such as granulocyte–macrophage colony‐stimulating factor, interleukin‐22 (IL‐22), IL‐23 and IL‐17, which orchestrate both innate and adaptive antibacterial immunity.^51^, ^52^, ^53^, ^57^ Innate defences against infection have generally been considered ‘hard‐wired’ – a specific infection will elicit a defined, stereotypical immune response.^58^, ^59^, ^60^ There has been a gradual re‐evaluation of this thinking because a variety of studies have shown that the host's resident microbes shape numerous aspects of host defences to airway infection.

A number of recent studies have demonstrated that the microbiota enhances the initial innate response to lung infection by bacteria.^37^, ^42^, ^61^, ^62^ One study showed that microbiota‐depleted mice had significant defects in early clearance (6 hr post‐infection) of *Klebsiella pneumoniae* from the lung, compared with mice with a microbiota.^42^ This correlated with decreased production of the inflammatory cytokines IL‐6 and tumour necrosis factor‐*α*. Attenuated bacterial clearance was the result of reduced production of reactive oxygen species by alveolar macrophages decreasing bacterial killing by these cells. Administration of Nod‐like receptor (Nod1 or Nod2) ligands by oral gavage, but not intranasally, restored lung defences in microbiota‐depleted mice, demonstrating the importance of PRR signalling in these phenomena.^42^ Other studies have demonstrated that in mice without a microbiota (germ‐free) *K. pneumoniae* lung infection causes significantly higher rates of mortality than in mice with a microbiota.^62^ Similar defects in lung defences were also seen in a model of *Escherichia coli* lung infection.^63^ This suggests that microbes in the gastrointestinal tract can have a systemic influence on antibacterial defences at distal mucosal sites and this occurs through PRR activation.

A further study corroborated and extended this work by showing that the microbiota enhances host resistance to pneumococcal pneumonia.^37^ In this work, microbiota‐depleted mice had increased bacterial loads in the lung and spleen after intranasal infection with *S. pneumoniae*, compared with mice with a microbiota. As with the previous study, these defects in host defences were evident 6 hr into lung infection with *S. pneumoniae* and accelerated the mortality rate of microbiota‐depleted animals. In the absence of the microbiota, phagocytosis of *S*. *pneumoniae* by alveolar macrophages was reduced, suggesting that it was defects in these innate cells that lead to attenuated antibacterial defences. Pneumococcal clearance and cytokine responses in the lung were restored in microbiota‐depleted mice if they were administered faeces from mice with a normal microbiota before lung infection.^37^ From these studies it is clear that the bactericidal capacity of alveolar macrophages is regulated by signals from the microbiota and these long‐lived tissue‐resident cells are therefore constantly gauging and responding to the host's microbial environment.

The influence of the microbiota is not limited to regulating antibacterial immunity, work has now demonstrated that it also promotes antiviral defences in the lung.^41^ Microbiota‐depleted mice infected with influenza have higher viral titres in the lung, compared with mice with a microbiota. Similarly to defects in antibacterial immunity, there was reduced lung cytokine production during infection, specifically IL‐18 and IL‐1*β* which are produced downstream of inflammasome activation. In the absence of the microbiota, there was reduced dendritic cell migration from the lung to the mediastinal lymph nodes, which was associated with reduced T‐cell responses and reduced influenza‐specific antibody production. These defects could be rescued by administration of Toll‐like receptor ligands via the airway or intrarectally into the gastrointestinal tract. Within the microbiota, it was found that a group of neomycin‐sensitive bacteria are a sufficient stimulus to enhance antiviral defences in the lung.^41^ Collectively, the studies demonstrate that defences against respiratory infection by major bacterial and viral pathogens are fortified by the microbiota.

The fact that depletion of a neomycin‐sensitive population of bacteria in the microbiota was sufficient to reduce resistance to influenza infection suggests that not all members of the microbiota have an equivalent effect on extra‐intestinal defences to infection. This hypothesis is supported by a study showing that mice with segmented filamentous bacteria (SFB) in the gastrointestinal tract survive *Staphylococcus aureus* pneumonia better than those without.^61^ SFB are known to regulate T‐cell differentiation in the murine intestine, which protects against intestinal infection,^47^ but this new work demonstrates that SFB also play a wider role in enhancing extra‐intestinal host defences. After intranasal infection with methicillin‐resistant *Staphylococcus aureus*, SFB‐negative mice had higher bacterial burdens in the lung and spleen, compared with SFB‐positive mice.^61^ Mice colonized with SFB had increased numbers of IL‐22‐producing cells in the lung, a cytokine known to enhance epithelial barrier integrity and drive the production of antimicrobial peptides crucial for mucosal defences.^64^ The importance of this cytokine was demonstrated by experiments showing that addition of recombinant IL‐22 to SFB‐negative mice rescued defects in lung immunity caused by the absence of SFB, and neutralization of IL‐22 in SFB‐positive mice abrogated any differences between SFB‐positive and SFB‐negative animals. If SFB‐negative mice were made SFB‐positive by co‐housing with SFB‐positive mice, or by oral gavage with SFB‐positive faeces, their defences against staphylococcal pneumonia were enhanced.^61^ Hence, differing microbiota compositions can have a significant impact on the host's resistance to extra‐intestinal infection.

Administration of PRR ligands or bacteria via the oral and rectal routes suggests that distal microbial signals can regulate lung immunity.^37^, ^41^, ^42^, ^61^ In addition to these distal signals, local microbes from the upper airway have also been demonstrated to regulate lower airway immunity.^41^, ^65^ It has been shown that mice maintained in specific pathogen‐free (SPF) conditions are more susceptible to influenza infection than those maintained in non‐SPF conditions.^65^ A distinctive feature of the non‐SPF mice used in this study was the higher load of commensal bacteria in the upper airway with a common colonizer of the upper airway in non‐SPF mice being *Staphylococcus aureus*. Treatment of mice with this bacterium before influenza inoculation reduced lung damage during influenza infection. Mechanistically, this protection was due to the recruitment of monocytes from the blood, which subsequently differentiated into anti‐inflammatory M2 alveolar macrophages in the lung.^65^ These macrophages inhibit the recruitment of excessive inflammatory cells during infection and it is thought that this reduces tissue damage and mortality caused by influenza.

## Host resistance to systemic infection and the microbiota

The major portal of entry for most pathogenic microbes is the mucosa. Mucosal defences are highly effective at neutralizing and eliminating the majority of infectious threats the host encounters. Periodically, however, dangerous microbes can survive mucosal defences, gain entry into the circulation and then disseminate to non‐mucosal tissues throughout the body. In these circumstances the host relies on bloodstream defences and blood filtering organs such as the spleen and liver to scavenge invading microbes. As non‐mucosal organs and tissues are not directly exposed to the environment they are not thought to be colonized by a microbiota. Because of the lack of direct contact with live microbes, it has long been presumed that the microbiota does not influence these systemic defences. We now know that this assumption is incorrect and that the production and function of cells that constitute systemic defences is greatly influenced by the microbiota.^29^, ^30^, ^39^, ^66^, ^67^, ^68^

Neutrophils are a major innate cell population in the bloodstream and are produced in the bone marrow before release into the blood. Numerous studies have shown that microbiota‐depleted mice produce significantly fewer neutrophils than mice with a microbiota.^39^, ^67^, ^69^, ^70^ Reduced neutrophil production renders these animals more susceptible to systemic infection by numerous bacteria including *E. coli* and *Listeria monocytogenes*.^39^, ^69^ Studies have shown that recognition of bacteria and/or bacterial products from the microbiota by PRRs is the first step in driving this microbiota‐dependent increase in neutrophil production.^67^, ^69^ This PRR activation is not restricted to a single site, as PRR ligands in the circulation and at the mucosa are able to drive increased neutrophil production.^67^, ^69^ The signals required downstream of PRRs, in contrast, are less well defined. A number of studies have shown that IL‐17 is important, as are members of the colony‐stimulating factor family.^67^ In addition to neutrophils, the production of macrophages and monocytes in the spleen is also promoted by the microbiota through currently poorly understood mechanisms.^39^ Hence, one of the most fundamental decisions made by the immune system – determining the number of innate cells required to safeguard the host from microbial assault – is directly regulated by the microbiota.

It is not only the production of innate cells that is controlled by the microbiota, but also the functioning of these cells. It has been demonstrated that the ability of neutrophils to migrate into tissues in response to inflammatory signals is attenuated after microbiota depletion,^71^ as is their bactericidal activity.^29^, ^37^ Again, both of these phenomena require PRR activation mediated by Toll‐like receptors in the case of extravasation^71^ and Nod‐like receptors to enhance bacterial killing.^29^ Currently, the exact mechanisms of neutrophil killing promoted by the microbiota are unknown. Cytokine production by systemic macrophages and dendritic cell populations is also regulated by the microbiota.^68^ Reduced cytokine production by these cells in the spleen, particularly reduced type I interferon, leads to attenuated host defences against systemic viral infection.^68^ During systemic lymphocytic choriomeningitis virus infection, this microbiota‐dependent enhancement of type I interferon production by dendritic cells primes a more robust NK cell response.^68^ This promotes viral eradication from the spleen. Again, the priming effect of the microbiota was mediated by PRR activation.^68^ Similarly, the microbiota enhances clearance of hepatitis B virus in the liver.^72^ This is thought to be due to signals from the microbiota enhancing both the cellular and humoral response to HBV infection. The mechanistic basis for this, however, remains to be determined.

## Host resistance to infection in the central nervous system and the microbiota

Alveolar and splenic macrophages are not the only macrophage populations under the influence of the microbiota. Microglia, the tissue macrophages of the central nervous system parenchyma, and the main innate cell population at this site,^73^ have also recently been shown to respond to signals from the microbiota.^74^ Their position in the central nervous system means that they are distal to sites of direct microbial colonization and hidden behind the protection of the blood–brain barrier.^73^ Microglia from mice without a microbiota have reduced expression of many genes connected to host defence including those involved in interleukin and mitogen‐activated protein kinase signalling pathways.^74^ These microglia also expressed high levels of CSF1, F4/80 and CD31, in comparison to microglia from mice with a microbiota. These markers are known to be down‐regulated as microglia mature, which suggests that the microbiota drives the maturation of these cells. As a consequence of this, in the absence of the microbiota, the microglia‐mediated response to inflammation and viral infection is defective. Reintroduction of the normal murine microbiota rescued defects in microglia function. Restoration of a limited number of bacterial species to mice without a microbiota, however, only partially rescued defects caused by lack of microbial stimulation.^74^ This suggests that complete immune maturation of the microglia requires a complex microbiota and/or specific bacterial groups within the microbiota. This complements studies of neutrophil production that have also shown that a complex microbiota is a more potent stimulus of neutrophil production than a microbiota of only a limited number of bacterial species.^69^ Short‐chain fatty acids, which are microbiota‐derived metabolites, were sufficient to restore microglia function in the absence of the microbiota, whereas loss of individual PRRs did not affect microglial activation.^74^ The maturation of immune defences in the central nervous system parallels recent work demonstrating that microbiota‐derived metabolites influences behaviour in mice.^75^, ^76^

## Conclusion

Our view of the role played by microbes in our health has been completely revised by recent studies of the microbiota. This change in perspective has refocused our thinking of microbes as purely disease‐causing threats to a more balanced perspective that incorporates microbes that can cause infection with those that play a beneficial and fundamental role in regulating many aspects of our normal physiology. Communication between microbes and host is mediated by the immune system. The immune system is now increasingly viewed, therefore, as a system whose normal role is to manage the microbiota day‐to‐day to exploit its beneficial effects, while guarding against the more occasional threats posed by infectious organisms.

From this increased understanding of how the microbiota influences host defences there are a number of core principles that are becoming apparent. (i) The effects of a microbiota colonizing a specific tissue are not restricted to that specific site. This is especially the case for the gastrointestinal tract, with bacteria at that site having an influence on nearly every tissue in the body. (ii) The interaction between the microbiota and immune system is highly dynamic. Many experimental studies have shown that depletion of the microbiota reduces the production and function of many immune cells, but restoration of the microbiota reverses many of these effects. The immune system is therefore constantly gauging the required degree of activation to efficiently manage the microbiota and pathogenic microbes without wasting resources. (iii) Pattern recognition receptors are constantly engaged by the microbiota and are the first steps in translating microbial signals into changes in host cell behaviour. Engagement of PRRs occurs directly at colonized environmental interfaces but can also occur in deeper non‐mucosal host tissues. PRRs are therefore being repositioned as homeostatic regulators of the immune system and not solely as sentinels of infection.^30^, ^43^ (iv) Not all members of the microbiota are equal in their ability to regulate the immune system. Members of the microbiota should not therefore be considered as simple packages of cell wall molecules that will all lead to equivalent pattern recognition receptor activation. (v) Tissue‐resident cells, exemplified by tissue macrophages, assimilate signals from both local and distal microbial populations. Furthermore, it is thought that in addition to the microbiota, infection and vaccination also leave lasting imprints on tissue‐resident macrophages.^30^, ^77^ The variety of different microbial encounters that the host experiences over its life‐course therefore defines the ‘set‐point’ of innate cell immune activation of a given tissue. This could, in part, explain the large environmentally driven variation in immune cell number, responses to cytokines, and vaccination found in humans.^78^

Despite rapid progress in understanding how the microbiota helps to protect against infection there are still many things that remain unclear. First, given that not all microbes regulate the immune system equally there is still limited information, apart from a restricted number of examples of local interactions in the gastrointestinal tract, on how different microbial groups influence various arms of host defence. A deeper understanding of how different members of the microbiota interact with innate immune receptors, host cells and what aspects of immunity they regulate is crucial to be able to fully harness the power of the microbiota for therapeutic benefit in the future. Second, only limited progress has been made in defining the signals and cellular functions that are influenced by the microbiota downstream of PRR activation. Third, it is still poorly understood how the microbiota helps protect against extra‐intestinal infections in humans. Some studies in humans have shown that changes in the microbiota are associated with changes in lung function and asthma;^79^ however, the specific effect on host resistance to lung infections remains unclear. By extension we have little understanding of how disruption of the microbiota by antibiotics at clinically relevant doses and durations influence host defences outside the gastrointestinal tract. Most mechanistic studies aimed at defining how the microbiota influences different aspects of systemic immunity have used prolonged treatment with broad‐spectrum antibiotics to deplete the microbiota. Although this is of great experimental utility, it provides limited information on how the microbiota disruption we experience when we are given antibiotics to treat infections affects immune function. Finally, as vaccination is the other major human intervention, along with antibiotics in the battle against infection, a more complete understanding of how the microbiota regulates vaccine responses could identify new ways to improve our defences against infectious disease.

## Disclosures

The authors declare that they have no competing interests.
